# *PvaPy* streaming framework for real-time data processing

**DOI:** 10.1107/S1600577525002115

**Published:** 2025-04-25

**Authors:** Siniša Veseli, John Hammonds, Steven Henke, Hannah Parraga, Barbara Frosik, Nicholas Schwarz

**Affiliations:** a Argonne National Laboratory 9700 South Cass Avenue Lemont IL60439 USA; RIKEN SPring-8 Center, Japan

**Keywords:** data streaming, real-time data processing, computing frameworks, *PvaPy*, *EPICS pvAccess*, Python applications

## Abstract

User facility upgrades, new measurement techniques, advances in data analysis algorithms as well as advances in detector capabilities result in an increasing amount of data collected at X-ray beamlines. Some of these data must be analyzed and reconstructed on demand to help execute experiments dynamically and modify them in real time. In turn, this requires a computing framework for real-time processing capable of moving data quickly from the detector to local or remote computing resources, processing data, and returning results to users. In this paper, we discuss the streaming framework built on top of *PvaPy*, a Python API for the *EPICS pvAccess* protocol. We describe the framework architecture and capabilities, and discuss scientific use cases and applications that benefit from streaming workflows implemented on top of this framework. We also illustrate the framework’s performance in terms of achievable data-processing rates for various detector image sizes.

## Abstract 2

A computing framework for real-time analysis of X-ray detector data streamed directly to Python applications via the EPICS pvAccess protocol is described.

## 1. Introduction

Large scientific user facilities, such as the synchrotron and free-electron laser light sources, can help to solve some of the most challenging scientific problems, ranging from efficient energy storage and transportation to the design of new materials for different purposes, to understanding the structure and motion of protein molecules. In search of answers to these problems, and aided by facility upgrades, technological advances in detectors, new measurement techniques, multi-modal data utilization and advances in data analysis algorithms, user experiments will generate larger and larger amounts of data. For example, after recent completion of its accelerator upgrade (Borland *et al.*, 2018 ▸), the combined output of X-ray beamlines at the Advanced Photon Source (APS) is expected to exceed 100 PB of uncompressed data per year, generated at over 100 GB s^−1^ (Carder *et al.*, 2022 ▸; Schwarz, 2022 ▸). The same trend of increased data generation volumes and rates has been observed at other similar new or upgraded experimental facilities (Schwarz *et al.*, 2020 ▸). By the end of this decade, the combined total of data generated across all the US light sources is estimated to be in the exabyte range, the processing of which will require large amounts of computing resources. It is clear that scientific discovery on this scale will present numerous challenges for data management and data analysis, as well as for the integration of user instruments and applications with storage, networking and computing resources.

At APS beamlines most of the existing production data analysis workflows are file based. Data are either written directly by X-ray detectors to the APS central storage system or are copied there from local beamline storage by the APS *Data Management System* (Veseli *et al.*, 2018 ▸). From there raw data files are copied using *Globus *(Foster, 2011 ▸) to the storage system visible to Polaris supercomputer nodes at the Argonne Leadership Computing Facility (ALCF) (Parraga *et al.*, 2023 ▸). After processing the raw data files, the results are typically copied back to APS storage in order to be visible to beamline scientists and facility users. The file-based workflows usually work well in cases where data analysis is not strictly time constrained, but they do come with overheads related to scheduling, executing and monitoring file transfers, scheduling batch jobs, as well as with file I/O operations required by data-processing applications.

Given continually increasing data volumes collected at experimental facilities, it is becoming increasingly important to analyze some of the collected data immediately, and provide feedback that can be used to adjust experiments in real time. This is especially significant for various machine-learning (ML) approaches for data processing and experiment control (Liu *et al.*, 2021 ▸; Yanxon *et al.*, 2023 ▸; Yanxon *et al.*, 2024 ▸; Babu *et al.*, 2023 ▸). For these cases where some or all the raw data must be processed in real time, there is a clear benefit to eliminating any unnecessary delays, including those associated with job scheduling, file transfers and file I/O.

Since a large percentage of X-ray beamlines use the *Experimental Physics and Industrial Control System* (*EPICS*) software (Dalesio *et al.*, 1991 ▸; Dalesio, 1993 ▸; Dalesio *et al.*, 1994 ▸) together with the *areaDetector* application (Rivers, 2024 ▸), this opens the possibility of developing *EPICS*-based streaming workflows for processing detector data in real time. Here we discuss the *PvaPy* streaming framework (https://github.com/epics-base/pvaPy/blob/master/documentation/streamingFramework.md), which allows users to set up distributed Python-based analysis workflows with very little effort, and without having to worry about details related to data serialization, networking protocols or integrating external software packages. We describe various framework components, user interfaces and available command line utilities. We outline several potential use cases and workflow configurations, as well as provide measurements for throughput and metadata handling tests that illustrate system performance. Finally, we also discuss a number of analysis applications that utilize streaming workflows and demonstrate the benefits of using *PvaPy* for their implementation.

## 2. Streaming framework

### 2.1. Foundation

The *EPICS* software is a collection of open source tools, libraries and applications used to create a distributed soft real-time control system for a variety of scientific instruments, such as particle accelerators and telescopes. It is developed collaboratively by a large community of developers and is used worldwide by hundreds of companies, universities, government laboratories and other scientific institutions.

*EPICS* uses two different protocols for communication between hardware and software devices and client applications. The *Channel Access* (*CA*) protocol (Hill, 1990 ▸) has been a part of *EPICS* since its beginning and is still used in most software applications. The *pvAccess* protocol (*EPICS* v4 Working Group, https://github.com/epics-base/pvAccessCPP/wiki/protocol) has been developed more recently and its usage is slowly increasing. Among other things, the *pvAccess* protocol provides the foundation for a service-oriented architecture, the ability to exchange complex data structures between server and client applications, optimized data transfers, as well as support for high-level data and image processing via *EPICS* normative types (https://docs.epics-controls.org/en/latest/specs/Normative-Types-Specification.html).

The *areaDetector* application provides a general-purpose interface for controlling 2D detectors using *EPICS*. It supports a wide variety of detectors and cameras and its architecture allows for processing images via a large number of included plugins. One of the included plugins, the *pvAccess Server* plugin, serves image data and its attributes over the *pvAccess* protocol using *EPICS NTND Array* normative types. The ability to retrieve images directly from this plugin using standard *EPICS* application programming interfaces (APIs) and command line interface tools is one of the major advantages of using *pvAccess* for streaming image data.

*PvaPy* (Veseli, 2015 ▸) uses the *Boost* Python library (https://www.boost.org) to provide a Python interface to *EPICS* C++ APIs. Some of its main C++-based features include:

(i) Support for both *CA* and *pvAccess* protocols used by *EPICS* software.

(ii) Support for scalars, structures and unions, as well as for *EPICS* Normative Types.

(iii) Integration with Python *NumPy*.

(iv) *pvAccess* server, including its *Data Distributor* plugin (https://github.com/epics-base/pvaPy/blob/master/documentation/dataDistributorPlugin.md), which enables several modes of distributing *EPICS* channel data to multiple client applications.

(v) *CA* input/output controller (IOC). This allows hosting standard *EPICS CA* database records directly from Python applications.

(vi) *Mirror* server, which is used for isolating data streams from clients that are not part of a particular workflow, for IOC protection from high client loads, or as a bridge between different networks or network subnets.

(vii) Support for channel and multichannel monitoring and client operations.

(viii) *Remote Procedure Call* (*RPC*) server/client. Unlike standard *EPICS pvAccess* servers, *RPC* servers do not support get/put/monitor operations on *pvAccess* channels, but instead provide client applications with the ability to invoke remote operations that take *pvAccess* structures as input.

### 2.2. Components

**Figure 1:**
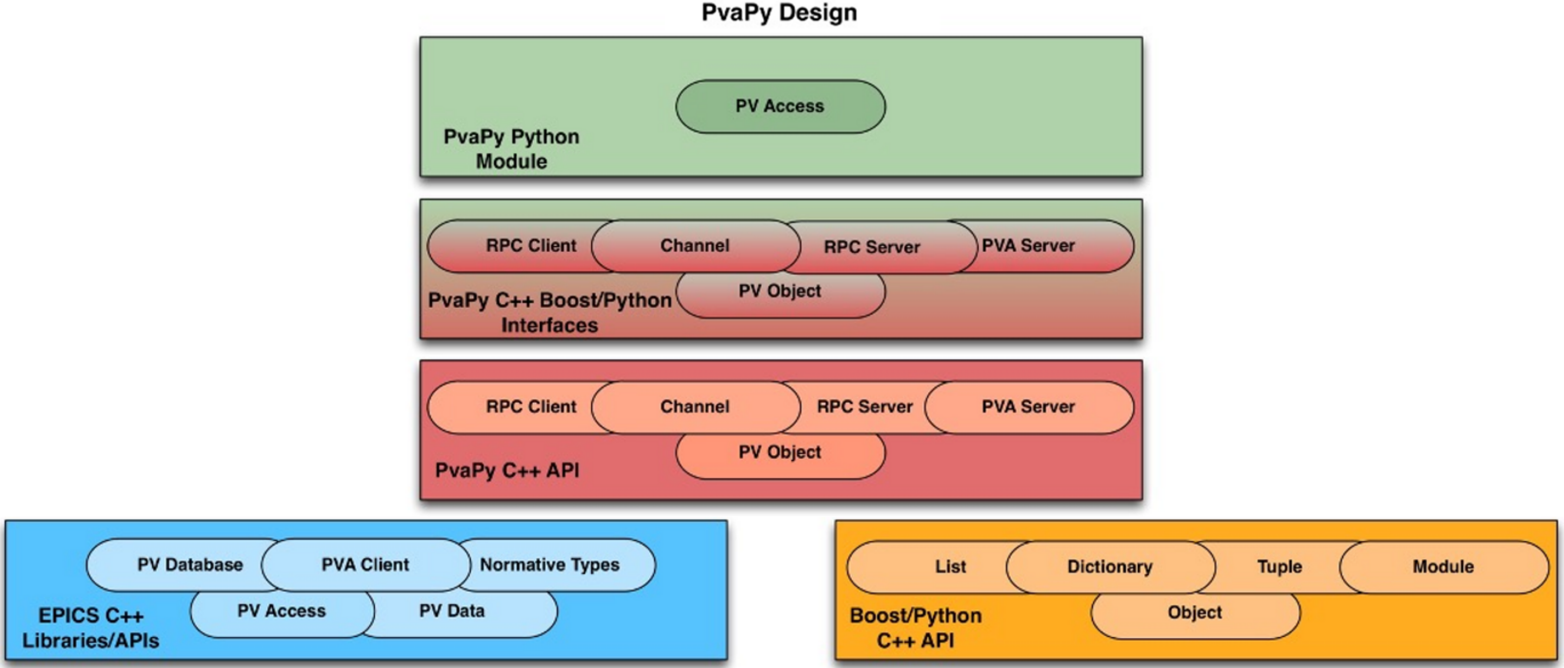
*PvaPy* uses *Boost* Python to wrap *EPICS* C++ APIs and provide corresponding functionality in Python.

The *PvaPy* streaming framework is written directly in Python and relies on its C++-based components for consuming and serving *EPICS* channel data (see Fig. 1 ▸). At its heart are the data consumer and data collector controller classes which are responsible for handling all common aspects of a processing workflow, such as managing processes that instantiate and configure user processing code, establishing input channel monitoring, providing workflow output channels, retrieving application statistics, invoking user processing code, and publishing its output. The two main command line interfaces corresponding to the consumer and collector controllers are *pvapy-hpc-consumer* and *pvapy-hpc-collector*. The consumer command is used for splitting input network streams and for processing stream data objects, whereas the collector command enables the user to gather data streams that were previously split for processing. In addition to those commands, the framework provides two commands that may be useful for constructing, developing and testing analysis workflows: pvapy-mirror-server can be used for data stream isolation and as a bridge between two different networks; and pvapy-ad-sim-server is an *areaDetector* simulator capable of generating and publishing *EPICS NTND Array* objects, and is typically used for testing and development of image-processing workflows.

**Figure 2:**
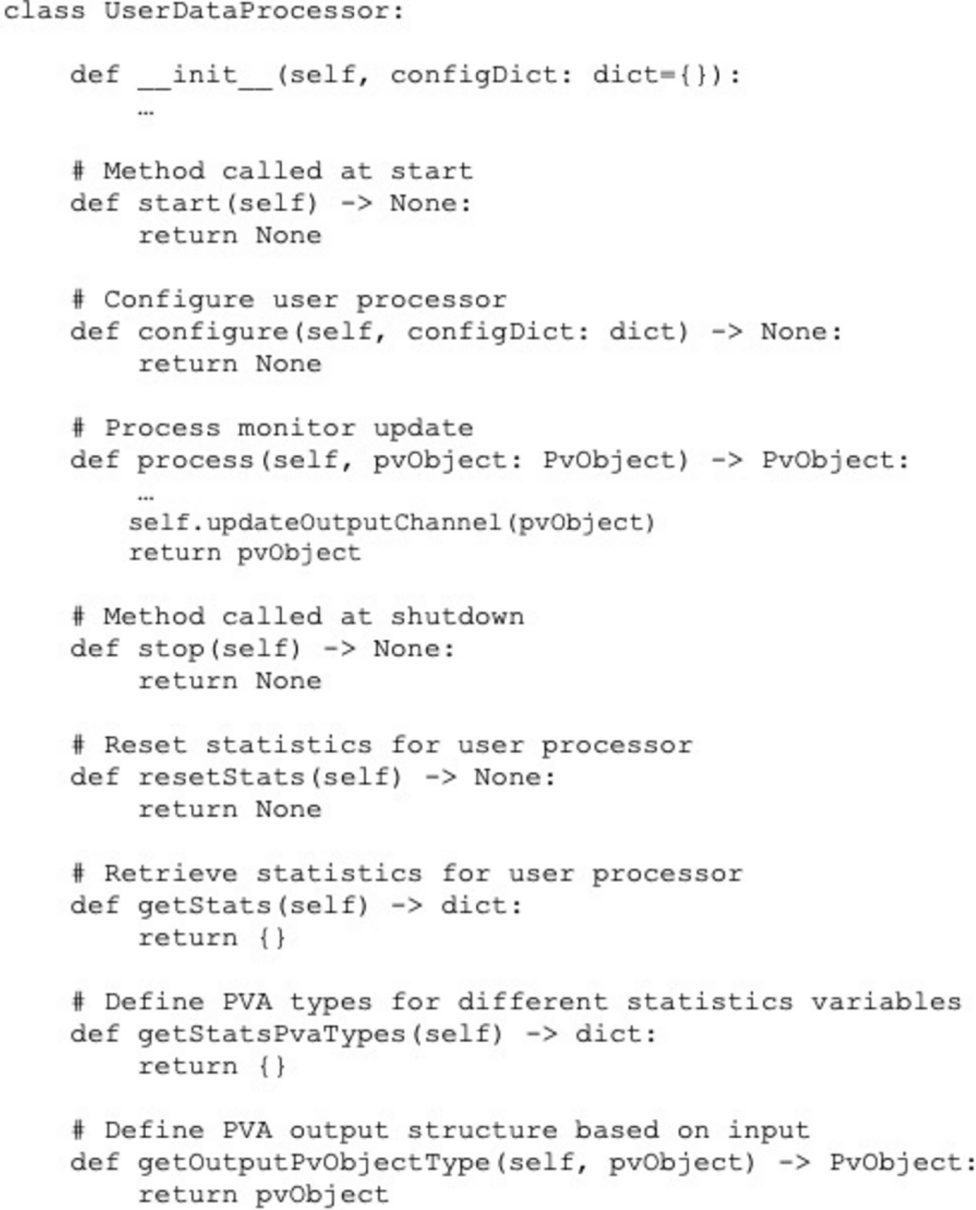
Streaming framework user interface hooks offered by the base processor class.

Users interface with the framework by implementing a data processor class, shown in Fig. 2 ▸. This class provides hooks called at different workflow stages, including methods for application startup and shutdown, runtime configuration, statistics generation, as well as the method for processing input channel data. The data processing method is called after the input channel monitor receives new data and is the only interface hook that must be implemented. From a framework perspective, a user application will see the data as a generic *EPICS* structure, which is similar to a Python dictionary. Applications that connect to an *areaDetector pvAccess Server* plugin receive data as *EPICS NTND Array*. In addition to the image data itself, this structure also contains image metadata that may be needed for processing, such as image dimensions and data type, color mode, compression algorithm, and other custom attributes. The user data processor class and its input, which is a *JSON* string representing a Python dictionary, are passed as arguments to the framework command line interface, which then establishes all framework processes on a given node.

### 2.3. Features

**Figure 3:**
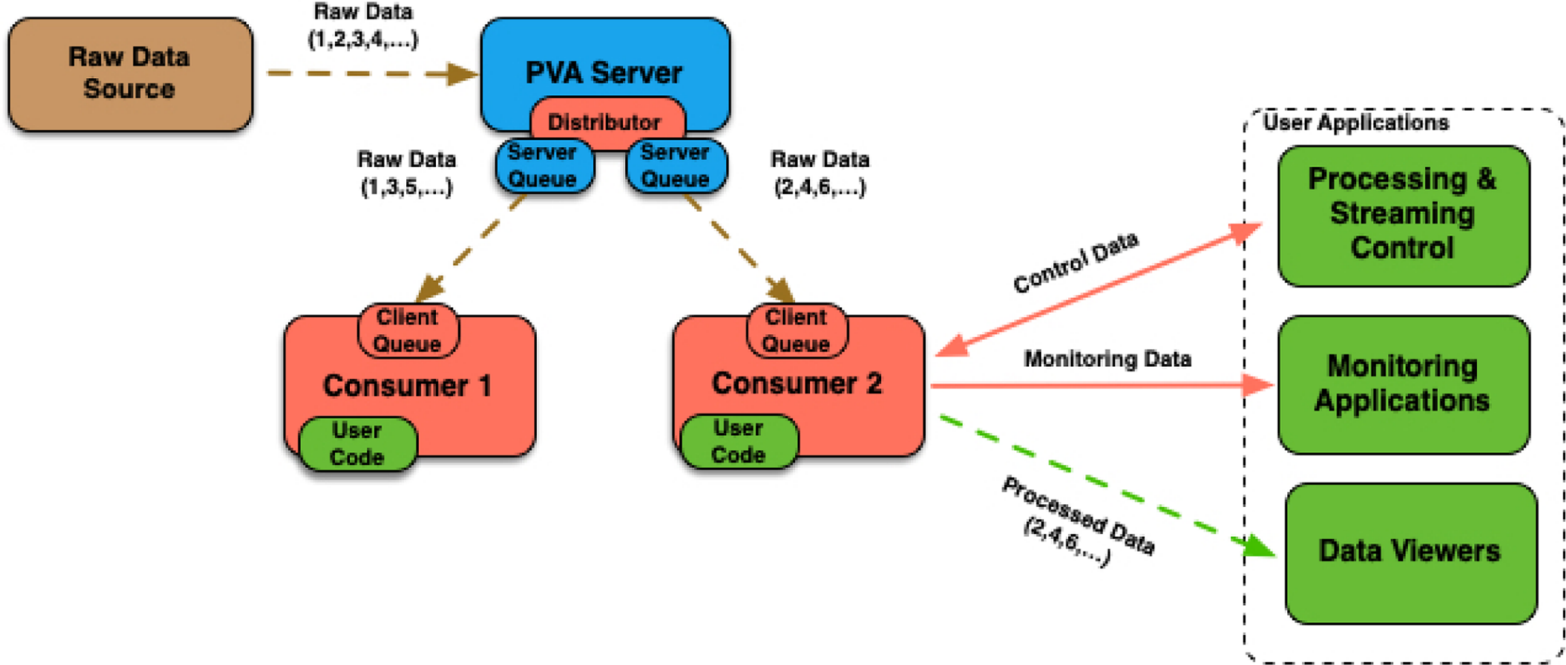
Streaming framework offers support for distributed processing, protection from lost stream objects, and the ability to monitor and interact with data consumer processes.

In addition to being straightforward to integrate and use, some of the other features offered by this framework are illustrated in Fig. 3 ▸ and listed below:

(i) Ability to receive and process generic *EPICS* structures as stream objects.

(ii) Support for a wide variety of workflow configurations, such as processing chains, and splitting and gathering streams.

(iii) Ability to receive metadata from additional *CA* or *pvAccess* channels that may be needed for processing main input stream objects.

(iv) Distribution of data processing between multiple consumer processes using the data distributor *pvAccess* server plugin.

(v) The system can easily spawn and manage multiple data consumer processes on a single node, and coordinate those with processes running on other nodes.

(vi) Runtime application configuration, monitoring performance and publishing output over additional *pvAccess* channels provided by the framework.

(vii) Protection against data loss via the server and client-side queues.

(viii) Support for data encryption. Note that the underlying *pvAccess* protocol does not support encryption as of the most recent *EPICS* 7.0.x release.

(ix) Standard *EPICS* utilities and APIs can be used for monitoring and interacting with the streaming framework.

Although *EPICS pvAccess* servers support limiting access to channels based on client usernames or hostnames, these features have not yet been implemented by the streaming framework. At the time of writing, *EPICS* and *pvAccess* do not support authentication methods, such as passwords or tokens. Any *pvAccess* client that has network access to systems serving channels created by the *areaDetector* application or by the data consumer processes, and knows the channel names and server port, could in principle connect to *pvAccess* data streams.

In the subsequent sections we expand on data distribution, data queuing capabilities and encryption support.

#### 2.3.1. Data distribution

Under normal circumstances all *EPICS pvAccess* or *CA* channel updates are served to all client applications. This mode of operation can become a problem when the network bandwidth is not large enough to send all updates to all clients continuously, or when data processing in client applications cannot keep up with channel updates. Real-time processing of *areaDetector* images generated at very high rates is one use case where these issues might occur. The *pvAccess Server Data Distributor* plugin solves these problems by enabling distribution of channel data between multiple client applications. The plugin considers two basic use cases for a group of clients:

(i) For simple parallel processing where client applications do not need to share data, all clients in a group receive *N* sequential updates in a round-robin fashion: client 1 sees the first *N* updates, client 2 the second *N* updates and so on.

(ii) For data analysis where several cooperating client applications must all see the same data to process, the applications are grouped into sets, and each set of clients receives the same number of sequential updates. The first *N* updates are sent to all members of client set 1, the second *N* updates are sent to all members of client set 2 and so on.

Parameters that determine how each client should receive data are configured during the initial connection via the channel request string. Different client groups are completely independent of each other, and the distributor plugin does not affect clients that do not explicitly request it.

#### 2.3.2. Protection against data loss

Both server-side and client-side queues can be arbitrarily large and can be used to smooth out variations in network bandwidth or application processing. The client-side queues are part of the *PvaPy* channel monitoring capabilities. They are initialized by the streaming framework based on user command line arguments and do not require any additional work by the user data processor class. The server-side queues are provided by the *EPICS pvAccess Server* and are established for each individual client upon connection. Those queues hold channel updates until the client can process the data, thereby providing protection against data loss that is only limited by the available memory of the system running the server.

#### 2.3.3. Data encryption

**Figure 4:**
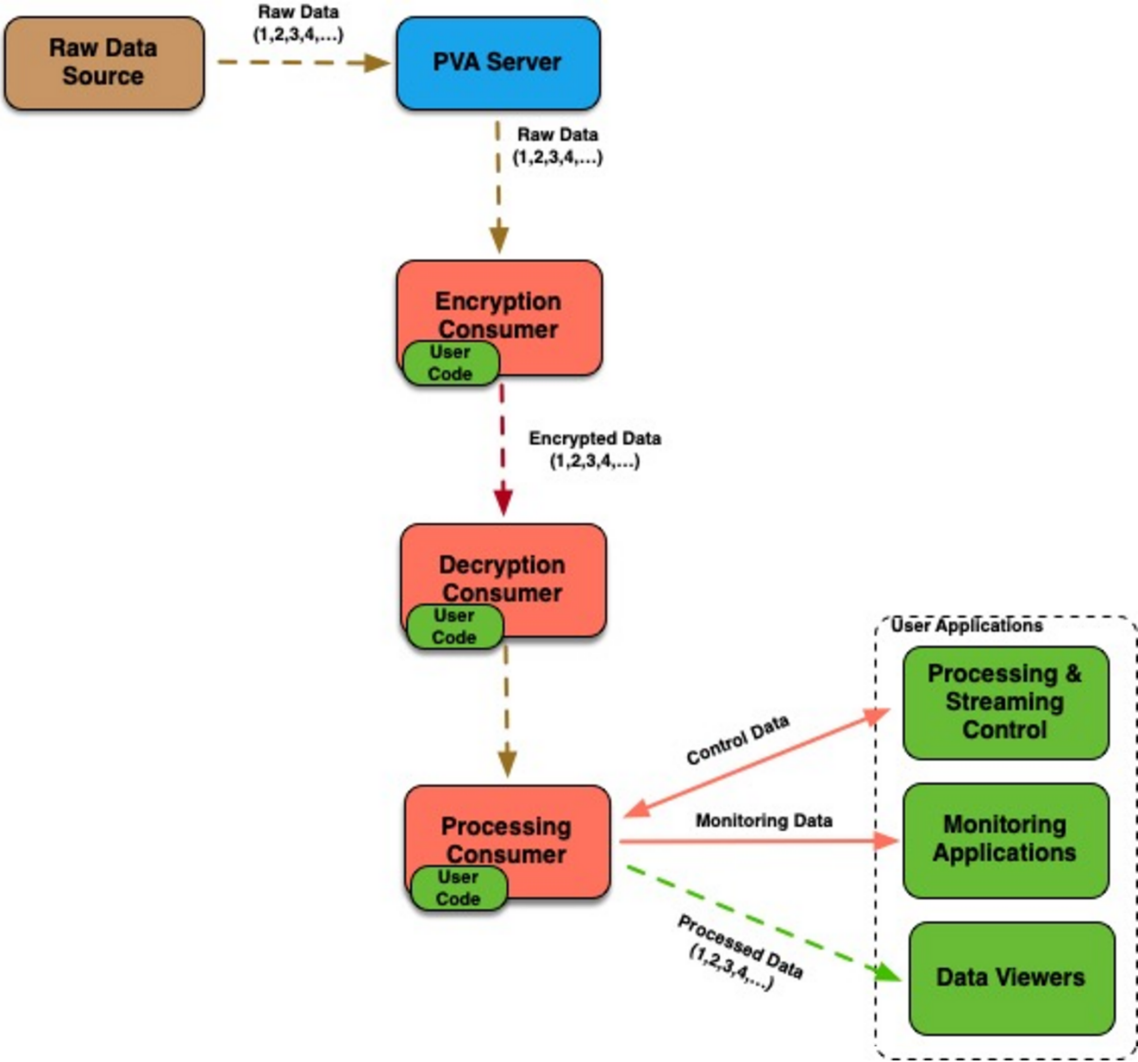
Streaming framework contains support for encrypting and decrypting generic *EPICS* data structures.

The streaming framework provides support for encrypting *EPICS* data structures before publishing them over *pvAccess*, as well as for decrypting received data structures before processing them. In case of *EPICS NTND Arrays*, the framework also provides encryption and decryption data processors that can be used out of the box. This allows one to construct workflows that work with encrypted data, even though following the *EPICS* release 7.0.x encryption is not yet available in the *pvAccess* protocol (see Fig. 4 ▸).

Placing the burden of encrypting and decrypting data on users rather than providing it as part of the protocol itself is certainly more complex, but it also does provide users with more control over where and how encryption takes place. This may be advantageous if there are performance concerns with a specific processing workflow, given that the encryption process adds computational overhead. For example, one might distribute data before encryption rather than encrypting it directly at the source, which would spread the encryption load between multiple systems. Another possibility would be to only encrypt sensitive parts of the data, which would reduce overheads.

A different approach for data encryption is to use SSH tunnels between machines running server and client-side applications. Although this approach generally requires no changes to user applications, it provides less flexibility as far as constructing processing workflows, and it can also raise potential security concerns related directly to SSH tunnels, such as uncontrolled port forwarding, network traffic obfuscation *etc*.

### 2.4. Comparison with alternative approaches

There are a plethora of available protocols optimized for streaming data, networking libraries and distributed messaging platforms that one could use for this purpose. Each of these choices has their own set of advantages and disadvantages. In terms of functionality and features, *ZeroMQ* (https://zeromq.org) is probably the most similar to the system described in this paper. It is a widely used embeddable networking library that also acts like a concurrency framework. *ZeroMQ* allows connecting sockets N-to-N with a variety of patterns like publish/subscribe, fan-out, task distribution, request/reply and client/server. As can be seen in Section 3, these patterns are very similar to workflow configurations that can be constructed with the framework described in this work. Much like *pvAccess*, *ZeroMQ* is designed for high throughput and low latency, but it also comes with native support for authentication and encryption, which is something that *pvAccess* protocol lacks at this time.

The main advantage that *pvAccess* has over *ZeroMQ* and any other choice for streaming data from detectors and sensors is its tight integration within the *EPICS* ecosystem. This integration makes the barrier to entry for *EPICS*-based applications relatively low when integrating with existing beamline software. It is easily deployed on beamlines that already use *EPICS*, and has the potential to ease reuse on other beamlines. There is no need to implement serialization and deserialization of data, write additional *areaDetector* plugins to publish data over a different protocol, or to write client applications for interacting with data, as would be the case when utilizing a different streaming tool.

## 3. Workflow configurations

One of the advantages of this streaming framework is its flexibility; it can be adapted to a wide variety of workflow configurations and analysis use cases. In this section we discuss some of the possible configurations and note that the framework documentation contains additional use cases as well as detailed framework usage and data processing examples that should work out of the box.

### 3.1. Processing chains

**Figure 5:**
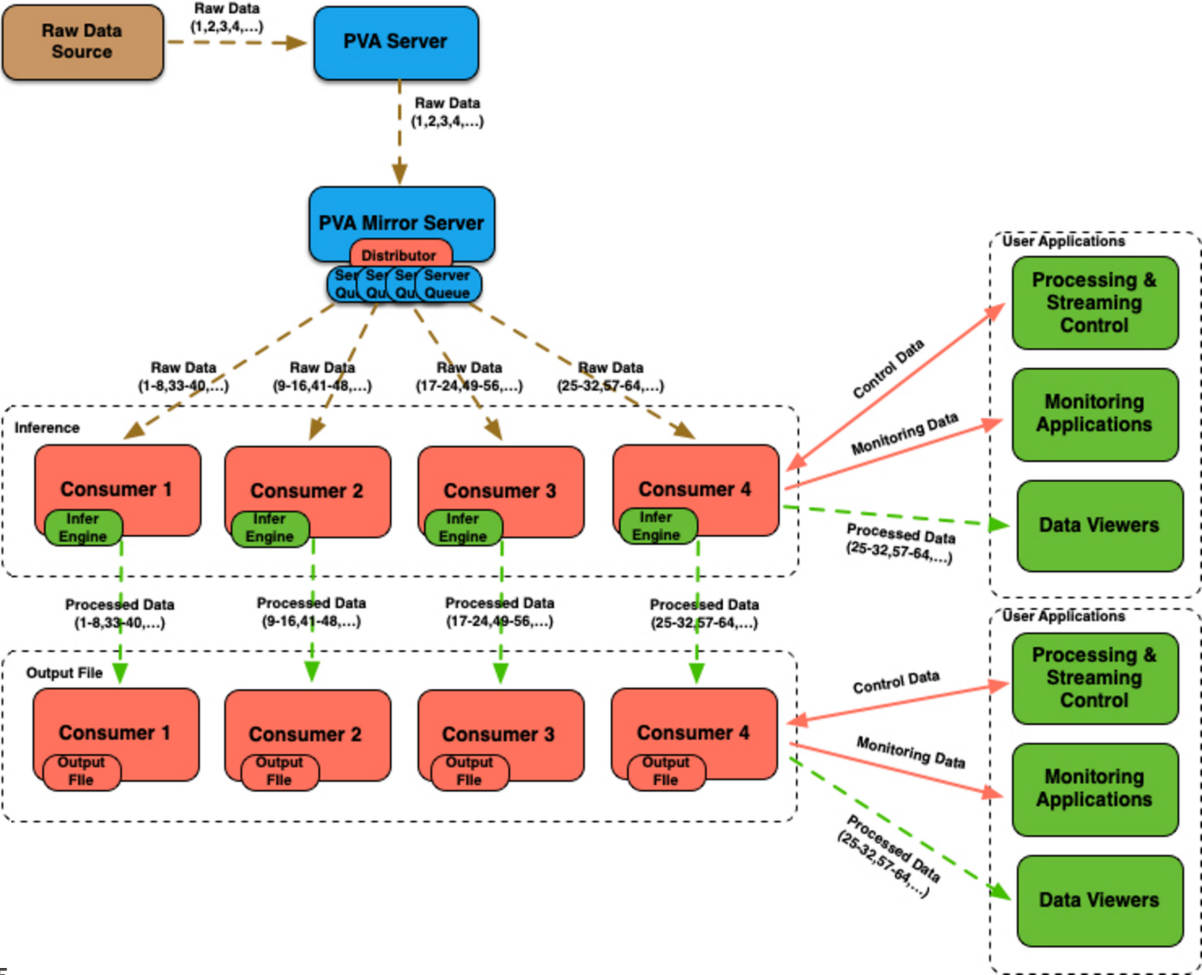
Example workflow that uses a processing chain configuration.

This workflow configuration demonstrates how stream processing can be implemented in multiple stages, with each stage running on a different computer or a set of computers (see Fig. 5 ▸). This is accomplished using the output of the first set of consumers as input to the second set of consumers. The *Mirror Server* is used for forwarding streamed objects from the source to the first set of consumers. This may be needed in case the raw data source (*e.g.areaDetector*) uses an older version of *EPICS* that does not have the data distributor plugin, or if consumers do not have direct access to the same network as the raw data source.

### 3.2. Splitting and stitching images

**Figure 6:**
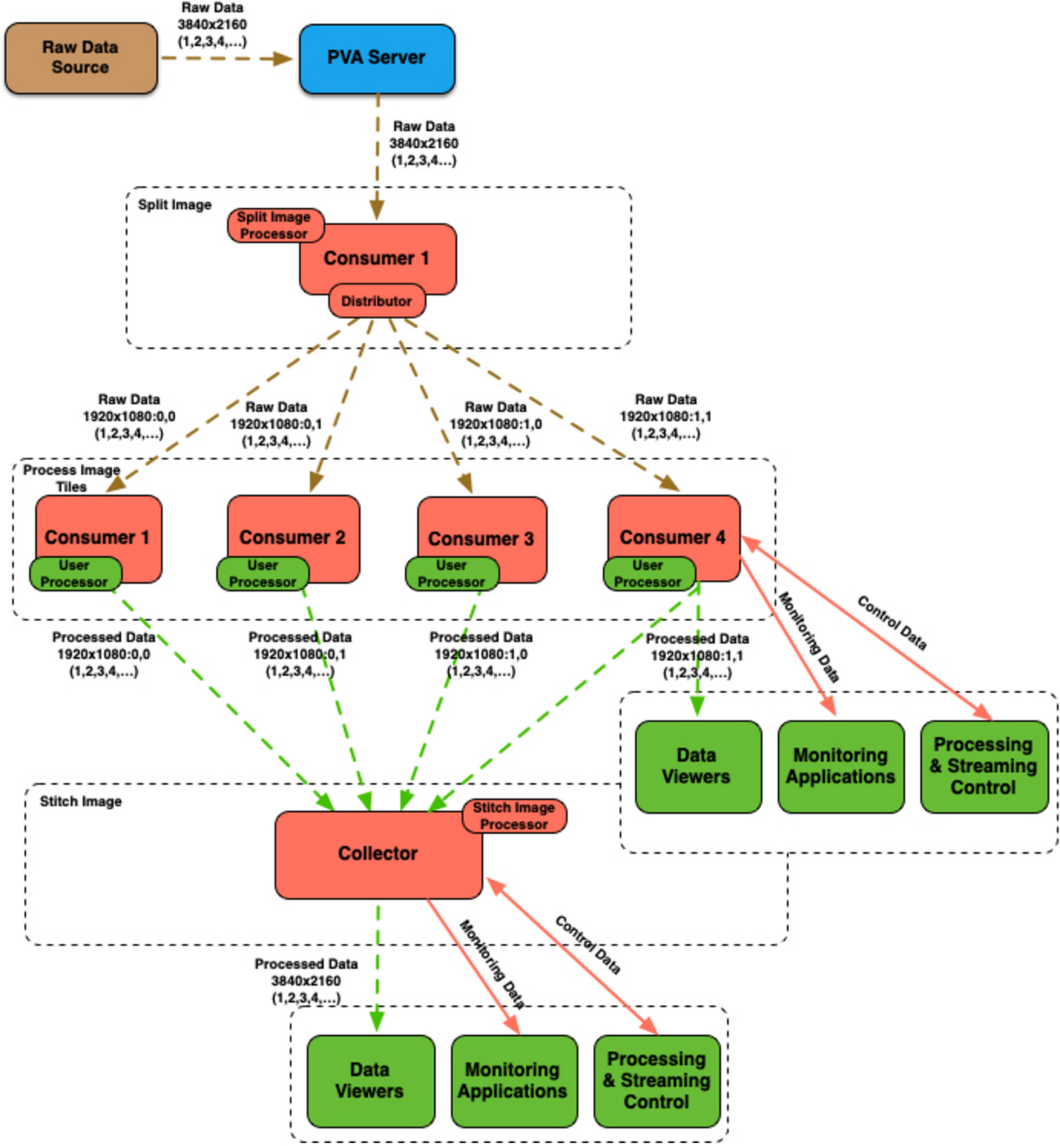
Example workflow for splitting and stitching images.

The example shown in Fig. 6 ▸ demonstrates how one can split the original raw image and distribute resulting tiles for processing between multiple consumers, and then stitch processed tiles back together. The last stage uses the data collector to gather streams resulting from processing of individual image tiles.

### 3.3. Image metadata handling

**Figure 7:**
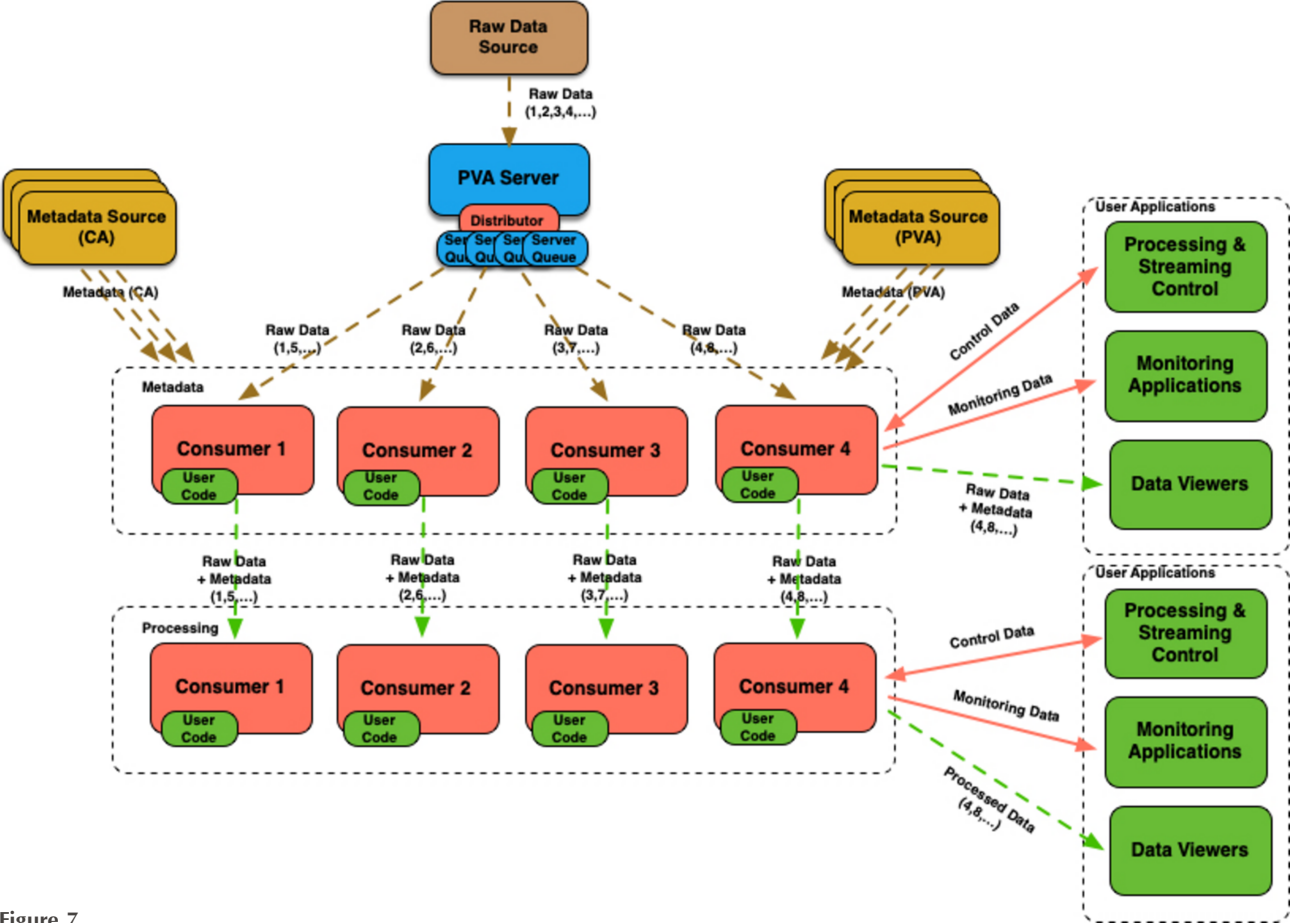
Example workflow for handling metadata available over *CA* or *pvAccess* channels.

In many cases images need to be associated with various pieces of metadata, such as position information, before processing. The streaming framework allows one to receive PV updates from any number of metadata channels, using *CA* or *pvAccess*, which are made available to the user processing module as a dictionary of metadata channel names and queues (see Fig. 7 ▸). Note that the streaming framework itself does not care what structure metadata channels produce, as anything that comes out of those channels is simply added to the corresponding metadata queues. However, the actual user processor must know the structure of the metadata object to make use of it.

## 4. System performance

All tests described in this section have been performed with *PvaPy* (version 5.4.1) on a 64-bit Linux computer with 96 logical CPU cores (Intel Xeon Gold 6342 CPU with hyperthreading enabled) running at 3.5 GHz, 2 TB of RAM and with a dual NVIDIA RTX A6000 GPU. Note that the image server and all consumers were running on the same computer using the loopback device. If these tests were performed using multiple computers, results might vary significantly depending on the network connection and configuration between the computers.

### 4.1. Throughput tests

To assess how much data can be pushed through the framework, we ran a series of tests using the base system, pass-through, user processor that does not manipulate images or encrypt data, and does not generate any additional load on the test system. We generated test images using the simulation server mentioned earlier, which is able to reliably generate images at stable rates of up to 26 kHz. Going beyond that number, the resulting output frame rate varied more than 1–2 Hz and was not deemed to be stable enough for testing. The server queue size varied with the test image size and was, in all test cases, set to keep less than a seconds worth of data per consumer. A given test was deemed successful if no frames were missed during the 60 s server runtime. Results for the maximum simulated detector rate that image consumers were able to sustain without missing any frames are shown in Tables 1 ▸ and 2 ▸ for 4096 × 4096 and 512 × 512 images, respectively. Note that the system was able to handle data throughput of more than 20 GB s^−1^ with larger images and a frame rate of up to 26 kHz with smaller images.

A similar set of tests was performed in the context of streaming unencrypted data from APS beamline computers to the ALCF Polaris supercomputer over a 200 Gbps network connection (Veseli *et al.*, 2023 ▸). For comparison with the results discussed above, the highest frame rate achieved in these APS/ALCF tests with 512 × 512 images was 24 kHz, and the highest data throughput observed with 4096 × 4096 images was 14.8 GB s^−1^.

### 4.2. Metadata handling tests

Metadata handling tests allow the assessment of how much unencrypted data can be pushed through the system in combination with metadata. Those tests used the sample *areaDetector* metadata processor module with six *pvAccess* metadata channels that were updated every time a new image was generated. A given test was deemed successful if no frames and metadata updates were missed during the 60 s server runtime, and if all images were associated with metadata without any errors. Results for the maximum simulated detector rate that image consumers were able to sustain and process are shown in Tables 3 ▸ and 4 ▸ for 4096 × 4096 and 512 × 512 images, respectively.

As the number of data consumers increases, the number of metadata updates that each consumer must discard increases as well, and hence gains in processing capabilities and in the corresponding data throughput decrease. This becomes more apparent with smaller images and higher frame rates; with 10 consumers the system handled a total of 4200 metadata updates per consumer per second for 4096 × 4096 images, compared with a total of 18000 metadata updates per consumer per second for 512 × 512 images. Some optimizations are achieved by batching sequential images received by consumers, as well as by reducing client load on the image data source via the *Mirror Server* or using parallel processing chains.

## 5. Applications

**Figure 8:**
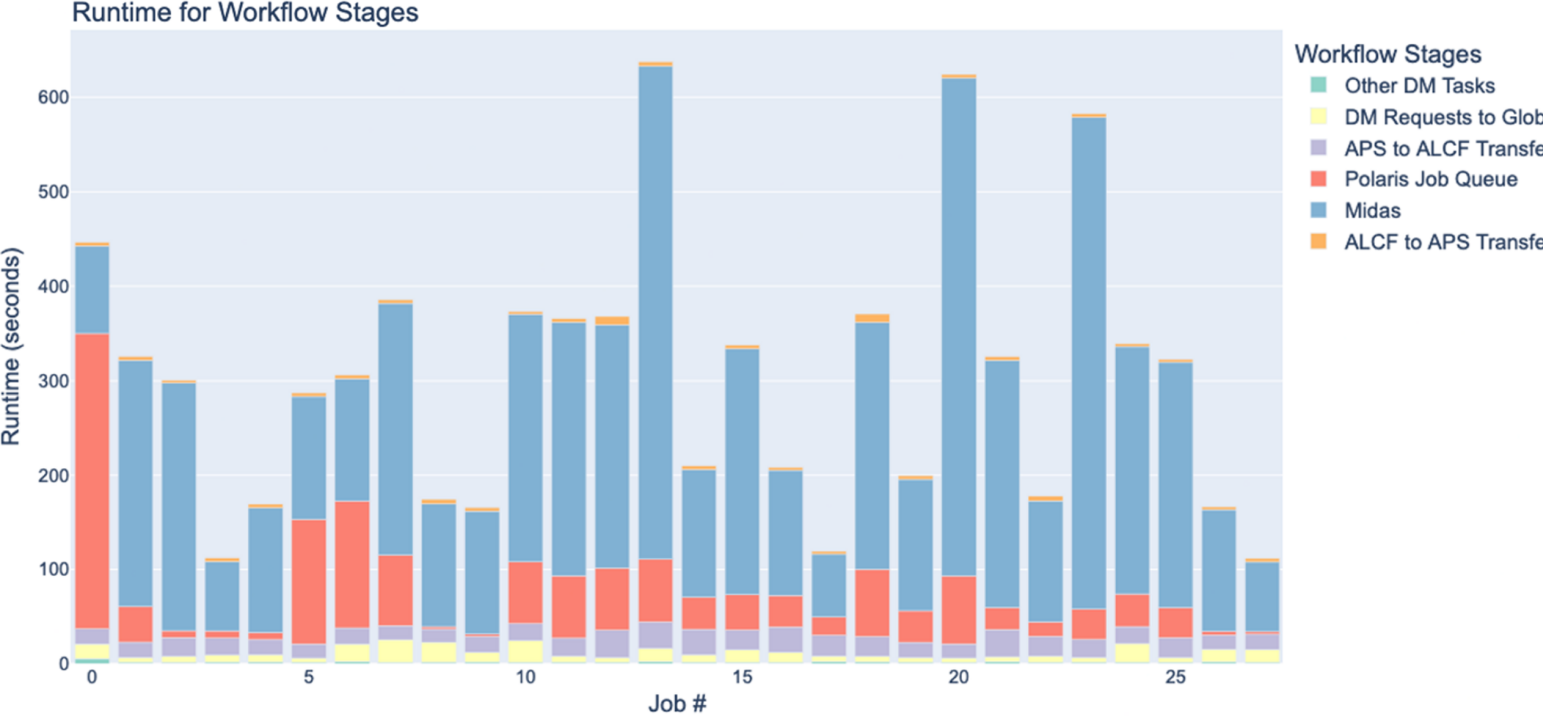
Execution runtime in seconds for different *MIDAS* workflow stages. The same raw dataset of 11 GB in size was processed by number of different jobs on the Polaris supercomputer.

Real-time feedback for dynamic experiment execution is becoming increasingly important, especially given the larger amounts of data being collected at X-ray beamlines. Streaming data directly from the detector into processing applications allows one to eliminate various delays typically associated with file-based analysis workflows. These delays are illustrated in Fig. 8 ▸, which shows execution times for different *MIDAS* (Sharma *et al.*, 2012*a* ▸; Sharma *et al.*, 2012*b* ▸) workflow stages in different Polaris supercomputer jobs processing the same input dataset. Because of their advantages in real-time processing, more and more streaming-based workflows are under development. For example, a solution for the real-time streaming tomographic reconstruction together with capability for 3D zooming to a volume of interest is presented by Nikitin *et al.* (2022 ▸). This helps users at the APS 2-BM beamline to set optimal environmental control system conditions, such as cooling temperature, pressure and loading forces, which is of crucial importance for experiments where the X-ray beam itself affects the sample state. It also allows users to overcome one of the main challenges in studying fast processes, the selection of a representative region of interest where dynamic processes start and evolve over time.

The work described above, as well as other applications discussed in this section, already use *PvaPy* for implementing streaming-related parts of their analysis workflows. The streaming framework brings the potential for significant performance improvements by distributing data to multiple application instances, the ability to use different streaming workflow configurations out of the box and reusing parts of the code common to most streaming workflows.

### 5.1. PtychoNN

One such example of streaming analysis involves *PtychoNN* (Cherukara *et al.*, 2020 ▸; Babu *et al.*, 2023 ▸), which uses a deep convolutional neural network to solve the ptychography phase retrieval problem. Phase retrieval algorithms are computationally expensive, which typically prevents real-time imaging. Using ML to predict a real-space structure and phase at each scan point solely from the corresponding diffraction data is hundreds of times faster than standard ptychography reconstruction packages and significantly accelerates data acquisition and analysis, which in turn has implications for the imaging of dose sensitive, dynamic and extremely voluminous samples.

**Figure 9:**
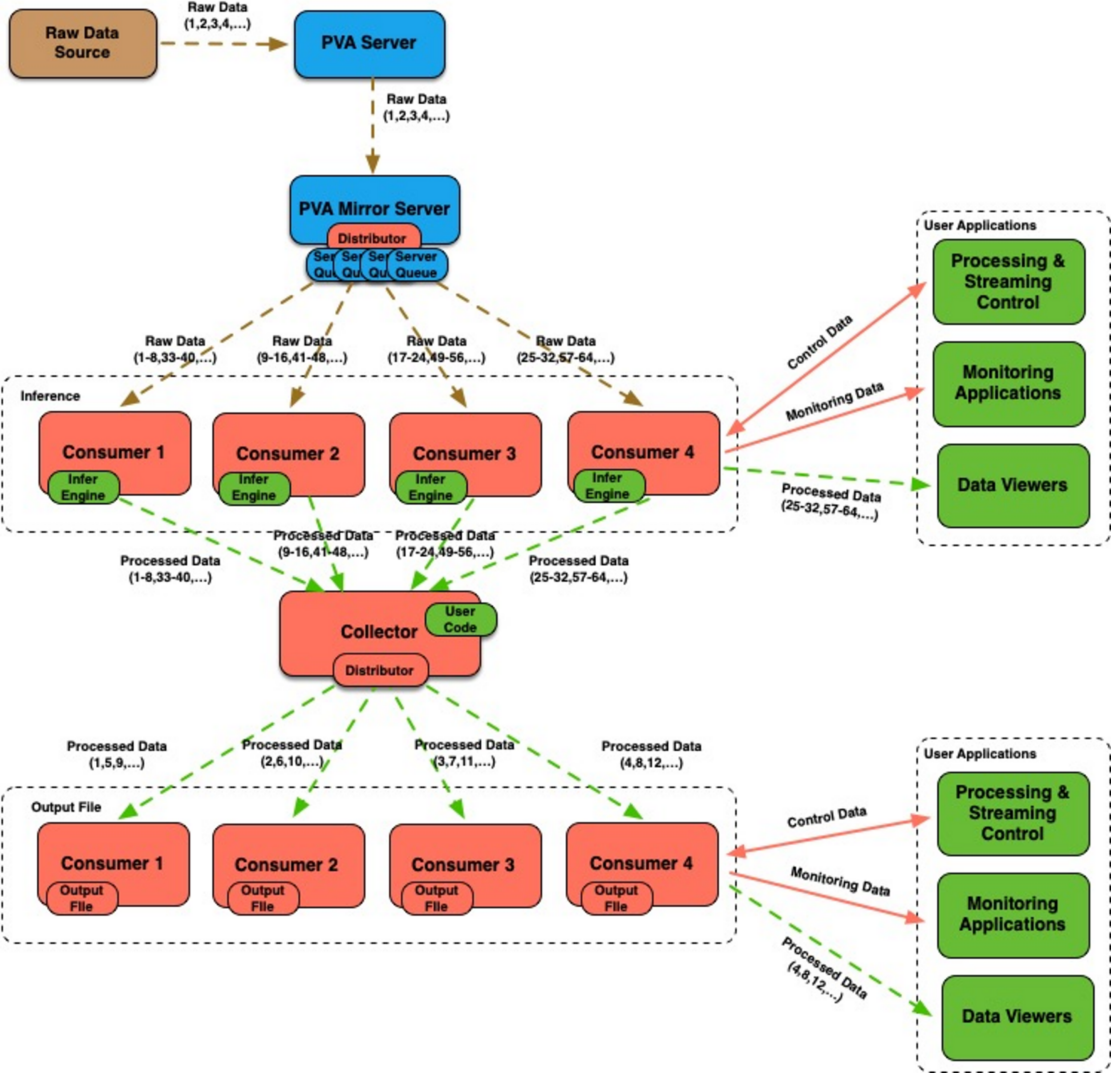
Streaming workflow used for *PtychoNN* processing (first set of four consumers) and for saving output frames to local storage (second set of four consumers). On a machine with a dual RTX A6000 GPU, the system was able to keep up with 8 kHz detector rates for 128 × 128 (int16) images without lost frames.

*PtychoNN* demonstrated real-time inversion capable of processing streamed raw 128 × 128 (int16, 32.77 KB) images at rates of up to 2 kHz on an NVIDIA RTX A6000 GPU (Babu *et al.*, 2023 ▸). After converting *PtychoNN* code to use the streaming framework (Veseli, 2024*a* ▸), the code-base was not only reduced by about 40% but the system was also capable of handling significantly higher data rates. When used in a mode where image processing is split between four consumers (two processes on each of the two RTX A6000 GPUs available, with each process receiving images in batches of eight) in a simple configuration illustrated in Fig. 3 ▸, we were able to keep up with detector rates of up to 8 kHz without any frame loss. The computer used for testing was the same one on which the results reported by Babu *et al.* (2023 ▸) were obtained. See Section 4 for more details. The system was able to handle the same detector rate even after adding a second set of four consumers in a processing chain configuration (see Fig. 5 ▸) that was responsible for saving processed images onto the local NVMe-based storage. At this 8 kHz detector rate, the system processed and saved 480000 images, about 15.7 GB of data, in 60 s. The same performance results were obtained with the *PvaPy Mirror Server* responsible for distributing data to the *PtychoNN* consumers, and the *PvaPy* data collector process responsible for collecting *PtychoNN* output and distributing it for processing to a second set of consumers (see Fig. 9 ▸). Since the processor used for saving files to local storage is already a part of *PvaPy*, there was no need for any additional user-supplied code.

**Figure 10:**
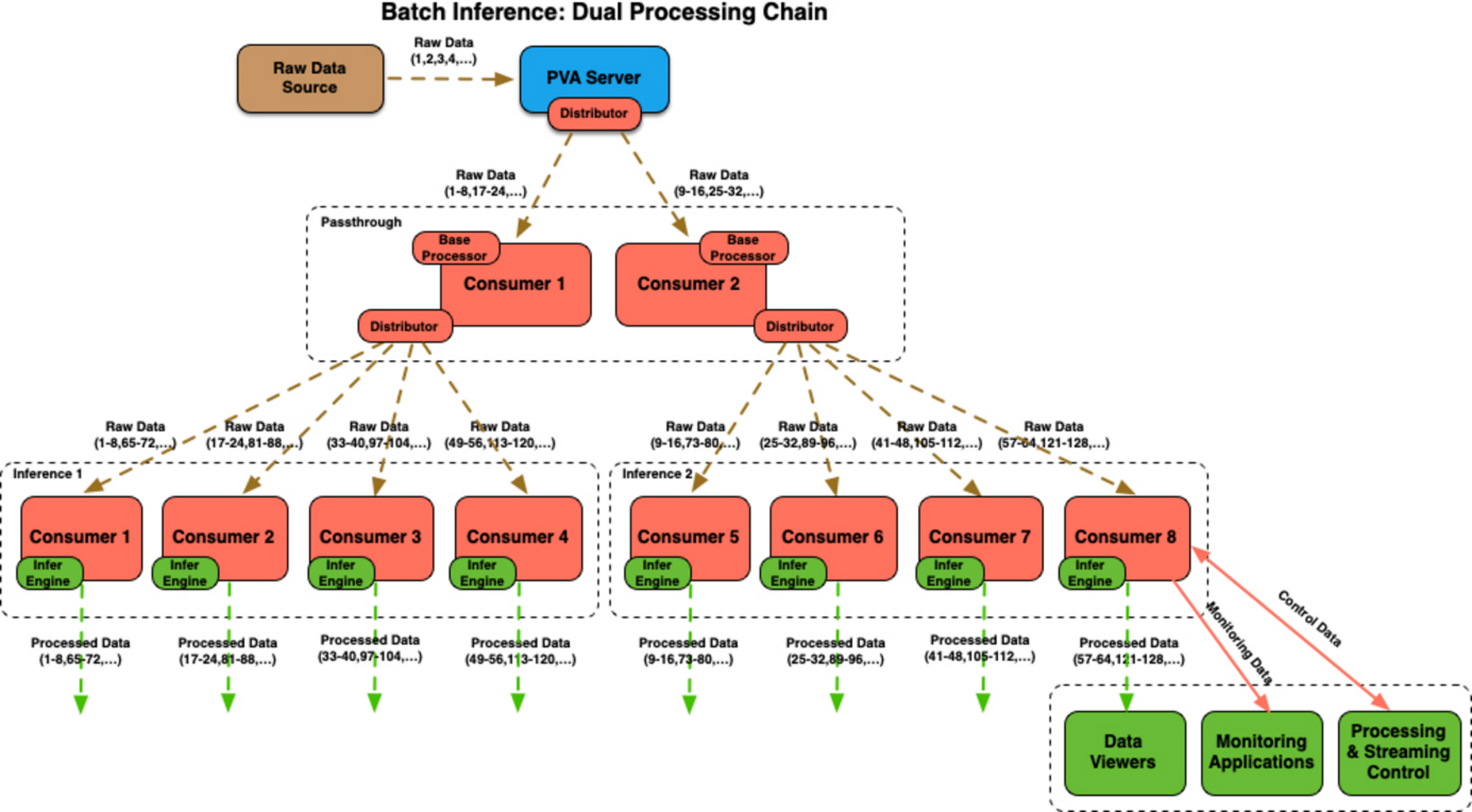
Streaming workflow used for *PtychoNN* processing via two parallel input streams (two sets of four consumers each). On a machine with a dual RTX A6000 GPU, the system was able to keep up with 12 kHz detector rates for 128 × 128 (int16) images without lost frames.

In a slightly more complex configuration, where the original raw data stream is split into two parallel streams using the pass-through data processor layer, and each of those then distributed processing between four *PtychoNN* consumers (see Fig. 10 ▸), the system was able to handle detector rates of 12 kHz without losing any frames. Note that in this case the pass-through layer split the 12 kHz raw data stream into two parallel 6 kHz streams, while each of the two RTX A6000 GPUs was running four *PtychoNN* processors receiving and analyzing images in batches of eight, and at a rate of 1.5 kHz.

### 5.2. BraggNN

Similar to *PtychoNN*, *BraggNN* is a deep-learning based method that helps to overcome computational costs associated with analysis of images obtained using high-energy diffraction microscopy (HEDM) (Liu *et al.*, 2021 ▸; Liu *et al.*, 2022 ▸). HEDM relies on knowledge of the positions of diffraction peaks, which are usually found by fitting the observed intensities in raw detector data to a theoretical peak shape such as Pseudo–Voigt (Bernier *et al.*, 2020 ▸). With increasing complexity of X-ray experiments, the computational costs associated with peak-shape fitting become an almost insurmountable obstacle for real-time analysis needed for the real-time feedback in experiments. The *BraggNN* approach has the potential to solve this problem and deliver significant performance improvements relative to conventional methods. The original *BraggNN* code was recently converted to use this streaming framework (Veseli, 2022 ▸), which offers similar capabilities and performance enhancements as those discussed in the context of *PtychoNN*.

### 5.3. Ptychodus

*Ptychodus* (Henke, 2024 ▸) is a ptychography analysis application that is used at several APS beamlines, which are predicted to be among the highest data producers after the recent upgrade of the APS accelerator. In order to help beamline scientists assess data quality in real time during acquisition, the streaming framework interface was added to *Ptychodus*. The streaming data processor receives diffraction pattern and scan position data streams directly from an instrument, and *Ptychodus* then uses timing information from the ingested data streams to robustly match diffraction patterns with their corresponding scan positions so the dataset can be reconstructed. The streaming workflow in *Ptychodus* is not yet production ready, as conventional iterative reconstructions cannot keep up with data acquisition speeds, and the workflow needs to be able to make use of multiple application instances reconstructing different scans in parallel. In addition, reconstruction algorithms based on neural networks and a continual learning process similar to those being used in *PtychoNN* (Babu *et al.*, 2023 ▸) are being added to *Ptychodus*.

### 5.4. Cohere

Bragg coherent diffraction imaging (BCDI) (Robinson & Harder, 2009 ▸; Harder & Robinson, 2013 ▸) is able to provide 3D information about internal strain, shape and lattice defects for nanometre-sized crystals. The *Cohere* toolkit (Frosik & Harder, 2024 ▸), developed in Python for the APS 34-IDC beamline, offers a complete solution for reading and pre-processing BCDI data, as well as for its reconstruction and visualization. The package design allows different software components to be used independently or as a whole. It also allows software to be customized for different instruments. Although at present *Cohere* is still aimed at file-based analysis workflows, the recent development work (Veseli, 2024*b* ▸) started adding streaming-based processing capabilities into the toolkit, with a future goal of using the *PvaPy* streaming framework for enabling real-time analysis of BCDI data.

### 5.5. Accelerator data processing

The recent APS upgrade (Borland *et al.*, 2018 ▸) will result in a significant increase in volumes of data produced not only at APS beamlines but also data produced by the new multi-bend achromat (MBA) storage ring accelerator. The new storage ring uses state-of-the-art embedded controllers coupled to various technical subsystems that have the ability to collect large amounts of data. The MBA *Data Acquisition System* (*DAQ*) (Veseli *et al.*, 2020 ▸) interfaces with a number of those subsystems to provide time-correlated and synchronously sampled data acquisition for statistics, diagnostics, performance monitoring and fault recording. For example, the storage ring radio frequency beam position monitors (BPMs) provide turn-by-turn (TBT) data that are collected by 20 TBT *DAQ* servers located around the storage ring accelerator. Each server receives beam position data from its TBT aggregator field-programmable gate array (FPGA) board. It generates a stream of *DAQ pvAccess* data objects at roughly 10 Hz. Individual double sector objects contain data for approximately 27000 turns obtained from 28 BPMs. In a default configuration the combined output of all *DAQ* TBT servers (560 BPMs) is about 95 MB s^−1^, which can be increased to about 245 MB s^−1^, if all available BPM data fields are collected.

**Figure 11:**
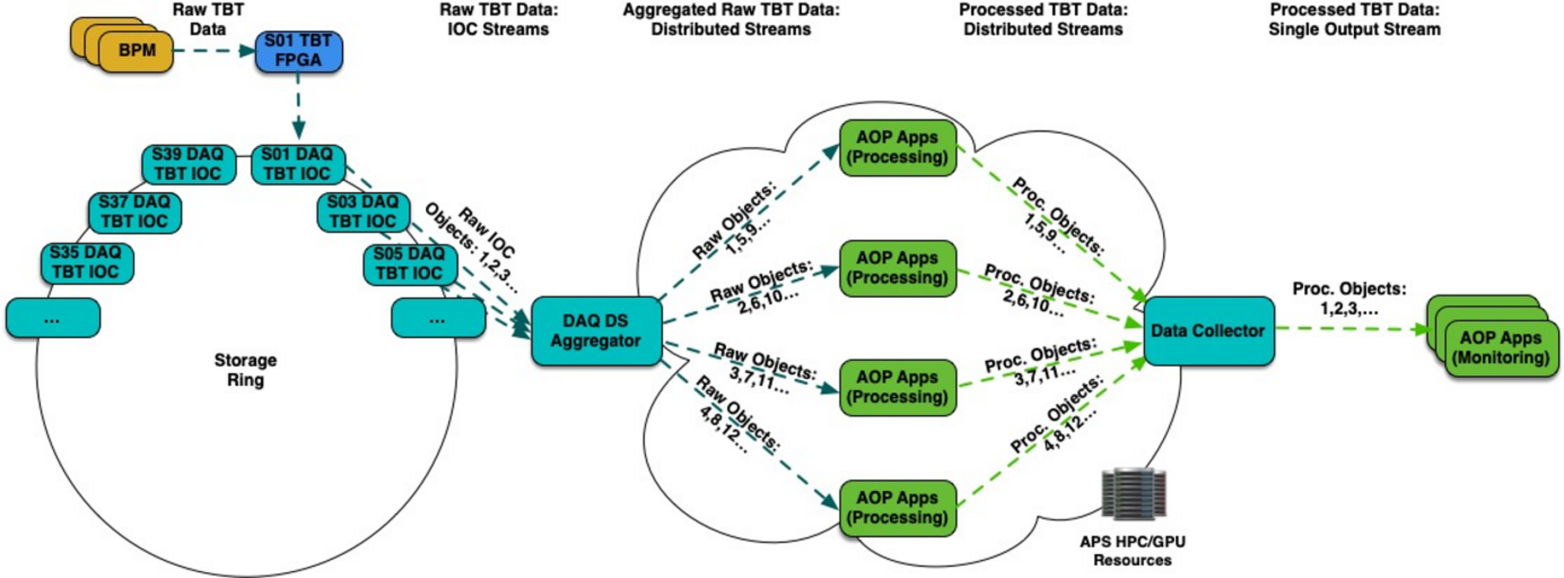
Distributed processing of the APS *DAQ* TBT data workflow. Raw BPM data around the storage ring are collected by the TBT FPGA and sent to the *DAQ* server, which publishes its raw *DAQ pvAccess* object. Individual *pvAccess* objects are combined into a full storage ring *DAQ pvAccess* object by the *DAQ* aggregator. The aggregator distributes processing of the raw storage ring *DAQ pvAccess* objects between a number of user application instances running as data consumers, and their individual output streams are combined and published as a single processed TBT stream by the *PvaPy* data collector.

In some use cases that require full TBT storage ring data using a single application instance for processing would not be able to keep up with the TBT data rates, and hence real-time analysis would have to be distributed between multiple application instances running on a local compute cluster. This workflow is illustrated in Fig. 11 ▸. The *DAQ Double Sector Aggregator* (Veseli, 2023 ▸) is a utility capable of collecting individual raw *DAQ pvAccess* objects from all servers, and combining them into a full storage ring *DAQ pvAccess* object. The stream of aggregated TBT objects can be distributed for processing between several application instances running as *PvaPy* data consumers and their individual output streams can be combined into a single output stream using the *PvaPy* data collector. One example application where distributed real-time processing of TBT data would be extremely useful is beam orbit generation, which is important for keeping the beam focused. The existing *DAQ Orbit* application (Veseli, 2020 ▸), which was heavily utilized during the machine commissioning and is now used during machine studies for accelerator performance tuning and diagnostics, cannot keep up with the full TBT data rate, and can only process up to a thousand turns at a 10 Hz rate. Implementing this application on top of the streaming framework is planned for the future.

## 6. Conclusions

We have discussed the *PvaPy* streaming framework developed for real-time processing of X-ray detector images, or any other types of objects being served over the *EPICS pvAccess* protocol. We have outlined framework features and illustrated possible streaming workflow configurations. The initial testing indicates very good performance with regard to data throughput. Using multiple pass-through consumer processes, the system has demonstrated the ability to handle frame rates of 26 kHz for small 0.26 MB images, and data rates of over 20 GB s^−1^ for large 16.78 MB images. We also discussed several applications that utilize streaming workflows and demonstrated advantages gained from implementing these workflows using the framework.

In addition to expanding the framework’s usage to different APS beamlines and scientific analysis applications, we also anticipate that work in the near future will focus on integrating the framework with systems that facilitate streaming between producers and consumers that lack direct network connectivity, such as *SciStream* (Chung *et al.*, 2022 ▸) and *EJFAT* (Sheldon *et al.*, 2023 ▸). The authors will also monitor *EPICS* developments regarding data encryption, authentication and authorization, and will consider them for implementation in this framework.

## Figures and Tables

**Table 1:** Maximum system throughput for 4096 × 4096 (uint8, 16.78 MB) images with *N* consumers Results are shown in terms of frames per second (fps) and data rates in gigabytes per second (GB s^−1^).

*N*	Frame rate/*N* (fps)	Frame rate (fps)	Data rate/*N* (GB s^−1^)	Data rate (GB s^−1^)
1	200	200	3.36	3.36
4	165	660	2.77	11.07
8	130	1040	2.18	17.44
10	125	1250	2.10	20.97

**Table 2:** Maximum system throughput for 512 × 512 (uint8, 0.26 MB) images with *N* consumers

*N*	Frame rate/*N* (fps)	Frame rate (fps)	Data rate/*N* (GB s^−1^)	Data rate (GB s^−1^)
1	16000	16000	4.19	4.19
4	6500	26000	1.70	6.82

**Table 3:** Maximum system throughput for 4096 × 4096 (uint8, 16.78 MB) images with *N* consumers and six *pvAccess* metadata channels, each updating with every new frame MD: metadata updates per second (mps).

*N*	Frame rate/*N* (fps)	Frame rate (fps)	MD rate/*N* (mps)	MD rate (mps)	Data rate/*N* (GB s^−1^)	Data rate (GB s^−1^)
1	200	200	1,200	1200	3.36	3.36
4	105	420	2520	10080	1.76	7.04
8	75	600	3600	28800	1.26	10.07
10	70	700	4200	42000	1.17	11.74

**Table 4:** Maximum system throughput for 512 × 512 (uint8, 0.26 MB) images with *N* consumers and six *pvAccess* metadata channels, each updating with every new frame

*N*	Frame rate/*N* (fps)	Frame rate (fps)	MD rate/*N* (mps)	MD rate (mps)	Data rate/*N* (GB s^−1^)	Data rate (GB s^−1^)
1	2000	2000	12000	12000	0.52	0.52
4	600	2400	14400	57600	0.16	0.63
8	350	2800	16800	67200	0.09	0.73
10	300	3000	18000	180000	0.08	0.78
